# Natural genetic variation in transcriptome reflects network structure inferred with major effect mutations: insulin/TOR and associated phenotypes in *Drosophila melanogaster*

**DOI:** 10.1186/1471-2164-10-124

**Published:** 2009-03-24

**Authors:** Sergey V Nuzhdin, Jennifer A Brisson, Andrew Pickering, Marta L Wayne, Lawrence G Harshman, Lauren M McIntyre

**Affiliations:** 1 Molecular and Computational Biology, University of Southern California, Los Angeles, CA 90089, USA; 2 University of Florida Genetics Institute, University of Florida, Gainesville FL 32610-36103, USA; 3 School of Biological Sciences, University of Nebraska at Lincoln, Lincoln, NE 68588, USA

## Abstract

**Background:**

A molecular process based genotype-to-phenotype map will ultimately enable us to predict how genetic variation among individuals results in phenotypic alterations. Building such a map is, however, far from straightforward. It requires understanding how molecular variation re-shapes developmental and metabolic networks, and how the functional state of these networks modifies phenotypes in genotype specific way. We focus on the latter problem by describing genetic variation in transcript levels of genes in the InR/TOR pathway among 72 *Drosophila melanogaster *genotypes.

**Results:**

We observe tight co-variance in transcript levels of genes not known to influence each other through direct transcriptional control. We summarize transcriptome variation with factor analyses, and observe strong co-variance of gene expression within the dFOXO-branch and within the TOR-branch of the pathway. Finally, we investigate whether major axes of transcriptome variation shape phenotypes expected to be influenced through the InR/TOR pathway. We find limited evidence that transcript levels of individual upstream genes in the InR/TOR pathway predict fly phenotypes in expected ways. However, there is no evidence that these effects are mediated through the major axes of downstream transcriptome variation.

**Conclusion:**

In summary, our results question the assertion of the 'sparse' nature of genetic networks, while validating and extending candidate gene approaches in the analyses of complex traits.

## Background

One of the frontiers of current day genomics is to build predictive models for how molecular variation in known pathways affects their performance. Here, we approach this question by focusing on one of the best mechanistically described processes. The insulin receptor/TOR kinase (InR/TOR) pathway underlies many physiological processes that redirect organismal resources into activity, maintenance, and survival, depending on dietary energy intake. Experiments performed on rats towards the start of the twentieth century identified dramatic increases in lifespan associated with a restricted diet [1]. Similar extensions in lifespan were subsequently identified in relation to dietary restriction in a broad range of other organisms, including the model organisms *Caenorhabditis elegans *and *Drosophila melanogaster *[2]. *C. elegans *has since served as a primary model for molecular descriptions of the InR/TOR pathway. Specifically, the tyrosine kinase receptor DAF-2 induces phosphorylation of the phosphoinositide 3-kinase (PI3K) catalytic subunit AGE-1 [3]. AGE-1 then phosphorylates the serine/threonine kinases AKT-1 and AKT-2, which form a complex with the serine/threonine protein kinase SGK-1 and subsequently phosphorylate the transcription factor DAF-16 [4-6]. The phosphorylated DAF-16 cannot enter the nucleus. Under normal dietary conditions, DAF-16 is retained in the cytoplasm in a phosphorylated state. However, if the receptor DAF-2 is inhibited, DAF-16 is not phosphorylated and so is capable of diffusing into the nucleus, where it regulates a range of genes that induce the starvation phenotype [7].

**Figure 1:**
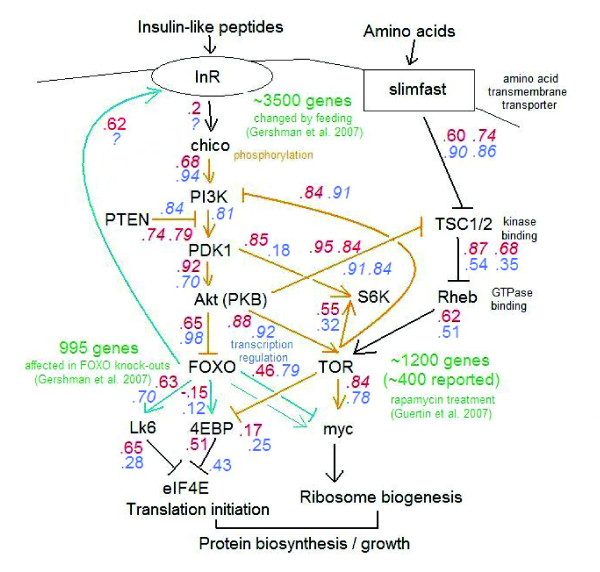
**The core connections in the InR/TOR network**. Regulatory connections through phosphorylation are shown in brown and through direct transcriptional regulation in blue. Arrows indicate activating interactions and bars inhibitory relationships. Correlation coefficients between genes are shown in blue for males and in red for females, with significant measures italicized; if there was no genetic variation for a particular gene, then the correlation could not be calculated and this is indicated by a '?'.

Recently, the InR/TOR signaling network was analyzed at the whole-genome level in *D. melanogaster *using microarray [8,9] and ChIP-chip analyses [10]. In Figure 1 we summarize these studies, along with other more specific studies querying regulatory relationships in the pathway. Mutations in the *Drosophila daf-2 *homologue *InR *present similar extensions in lifespan to *daf-2*, implying a similar function [11]. The forkhead transcription factor dFOXO is likely the functional equivalent of DAF-16 [12]. Insulin levels regulate *InR*, which signals to PI3K through *chico *– the gene that encodes a likely ortholog to *human substrate protein*. PI3K suppresses dFOXO by means of AKT-mediated phosphorylation, causing dFOXO to remain localized in the cytoplasm [13]. As a result, dFOXO cannot access its direct downstream binding targets, including 4E-BP and Lk6. In the absence of dFOXO binding, 4E-BP and Lk6 repress eIF4E, and thus repress translation initiation. PI3K also activates TOR, which in turn upregulates *myc*, one of the dFOXO downstream targets that affects ribosome biogenesis. These highly coordinated responses, which differ slightly among tissues, result in fine-tuned regulation of protein biosynthetic capacity. There are several feedback loops in this hierarchy as well, including that *InR *transcript level is affected by dFOXO itself through direct binding [14]. Thus, the InR/TOR pathway is interesting as it combines multiple levels of regulation, including both transcription and translation. However, whether the pathway's components are coordinated with each other at the level of transcript abundance remains unresolved.

In model species for genetic research, the InR/TOR signaling network structure and its phenotypic effects were studied via analyses of major effect mutations. It is unknown if smaller genetic alterations affect gene expression and phenotypes in an analogous manner. One type of smaller effect perturbation is genetic variation among natural genotypes, which in turn results in variation among gene transcript levels [15]. The most comprehensive recent data set of this type is by Wayne *et al*. [16] describing whole genome expression levels in 72 genotypes of males and females. Here we use this data set to investigate whether we can detect covariance in transcript variation in InR/TOR pathway genes. Because the high dimensionality of genome-wide expression data presents multiple challenges [17-21], we focused our analyses on three *a priori *defined groups of co-regulated genes. Gershman *et al*. [8] identified 3519 genes involved in the transcriptional response to nutrition in *Drosophila *by assaying RNA of adults that had been fed yeast following a period of starvation. They also identified a 995 gene subset of the nutrition-affected genes using microarray analysis of Drosophila S2 cells constitutively expressing dFOXO. Likewise, a list of 1016 genes downstream of TOR has been inferred via rapamycin treatment of Drosophila S2 cells [9]. Using the gene expression states across 72 genotypes described in Wayne et al [16], we examined how the patterns of variance/covariance in these gene expression levels reflect variation in the upstream InR/TOR network. Specifically, we wanted to determine whether or not gene expression levels showed tighter covariance among genes from the individual TOR or dFOXO branches of the pathways, relative to the pathway overall.

Given that thousands of genes can be perturbed at once by variation in only a few upstream genes, the question arises as to how to summarize variation of their expression levels into fewer gene clusters co-varying for expression among genotypes. Several dimensionality reduction approaches are available, including principal components, spectral map and correspondence analyses [22,23]. We focused on factor analysis, which uses covariation among genes to identify factors affecting the transcription of multiple genes at once [24]. One may interpret the factor as the mechanism, for example, a transcription factor, by which genes are co-regulated. Another example would be that of tissue-specific expression; genotypes with a larger volume of this particular tissue would have higher levels of all tissue-specific genes. Whatever the true underlying mechanism, the factor model represents sets of coordinately expressed genes. Taking this a step further, these covarying genes can be viewed as participating jointly in a network. Each gene has an estimate of its participation in the common network; this is called the factor load, where the strength of the load indicates how much that particular gene contributes to a given factor. The product of the factor load and the gene expression value for a given genotype can then be summed over the individual genes considered into a factor value. The factor value then represents the functional state of this network, *i.e*., how much the individual genes in the network are controlled by a common regulatory mechanism in each genotype. Our first goal was therefore to use factor analysis to investigate the variance/covariance structure of genes in the InR/TOR pathway.

Our second goal was to investigate the phenotypic consequences of natural genetic variation in the InR/TOR pathway, since major effect mutations in the pathway are known to affect lifespan, survival, and other life history characters [25-27]. Similar phenotypic effects of these mutations were previously assayed in flies with gross abnormalities of pathway functioning [e.g., [11,27]]. We know that knocking out the gene function of dFOXO results in higher desiccation resistance [28]. Will smaller, quantitative expression changes of dFOXO result in accompanying phenotypic modification or it will be buffered? Our goal was to determine whether natural quantitative variation in transcript levels of these genes would have quantitative effects on the phenotype resembling the effects of major mutations. We analyzed co-variation between transcript levels in the upstream InR/TOR pathway with candidate life-history associated phenotypes, and confirmed many patterns expected from previous analyses of major-effect mutations. Our findings extend the candidate gene approach as a helpful tool in the analysis of phenotypic variation.

The summary factor analysis of the downstream InR/TOR-affected transcriptome can also be examined for associations with phenotypic traits by correlating the estimated factors with the phenotypes [29]. This correlation serves to infer which factors, *i. e*., major axes of transcriptome variation, contribute to the modification of which phenotypes. By studying these correlations, we were unable to connect major axes of transcriptional variation in the InR/TOR to phenotypic variation. This observation questions the utility of this type of 'perturbation screen' for identifying *de-novo *axes of expression variation and simultaneously accounting for phenotypic variation.

## Results

### (i) Genetic variation-covariation between the upstream genes in the InR/TOR pathway

We have reanalyzed the data of Wayne *et al*. [16] to determine whether genetic variation and covariation in transcript abundance among genotypes reflect the structure of a known *D. melanogaster *network, the InR/TOR pathway. Wayne *et al*. [16] recorded microarray hybridization signals of whole body RNA extracted from heterozygous F_1 _male and female progeny obtained from all possible crosses between 9 genotypes from a single natural population, resulting in 72 replicated measurements of expression in each sex. Significant co-variation between gene expression levels of two or more genes in this dataset may be due not only to biology, but also to experimental artifacts. Linked alleles within a homozygous parental genome are always co-inherited in F_1 _progeny originating from this parent. F_1 _offspring sharing a parent may then exhibit patterns of association due only to the co-inheritance of the same block of alleles rather than true association among the loci in their underlying mechanism. In such a case, 9 genotypes out of 72 will have a high combination for their transcript levels, which is a pattern akin to pseudoreplication. To deal with this problem, instead of studying the patterns of covariance among the 72 genotypes, we first estimated breeding values for transcript levels in the 9 parental genotypes (Additional files 1 and 2) and determined the effect of breeding values on transcript levels in our subsequent analyses. We focused on the portion of the InR/TOR network that: i) is well established in flies, ii) genetically varies among the genotypes, and iii) is represented by high quality oligonucleotides on the custom microarray designed by McIntyre *et al*. [30]. Some genes were tagged by several oligonucleotides and all of them were retained and their signals averaged in the subsequent analyses.

The InR/TOR signaling network combines regulation at the level of transcriptional regulation, phosphorylation, protein relocation and other molecular mechanisms. Genes that are in the InR/TOR pathway and have been analyzed by this study are represented on Figure 1. We determined the covariance of transcript levels of these genes. Despite the diversity of molecular functions and mechanisms of signaling, the transcripts of nearly all genes in the network strongly co-vary with each other (see numbers on arrows, Figure 1). For instance, the genetic correlation between the transcript levels of AKT and TOR (AKT phosphorylates the TOR protein) are 0.88 in females and 0.92 in males, both highly significant. Clearly, this co-variation is not necessarily due to causal effects of the gene's transcript levels on one another, but is most likely due to the influence of other genetic factors onto both of these genes. This might indicate a common regulatory mechanism for the pathway as a whole within a cell. Alternatively, as tissue representation might vary among the genotypes and InR/TOR network function varies between tissues, the correlations might be due to these organism-level differences among the genotypes. Note that environmental or developmental fluctuations may be ruled out as explaining these patterns of co-variation: transcript level measurements in each heterozygous genotype were biologically replicated to account for dye and environmental effects. Further, an effect of each genotype (breeding value, see [31] for definitions) was estimated from a joint analysis of 72 heterozygous genotypes, further reducing the opportunity for developmental and environmental noise to influence the analyses.

While we only present correlation coefficients for the genes known to signal each other on Figure 1, we also estimated correlations between all pairs of these genes (Additional file 3). Overall, they represent a highly connected, co-varying group from which it is difficult to recover any fine structure. We conclude that, as expected, the genes of the InR/TOR network are strongly co-regulated at the cellular or organismal level, but the strength of genetic co-variations among them does not mirror the expectations based on the mechanistic details known for this signal transduction pathway.

### (ii) Genetic variation for the levels of message among feeding-affected genes

Gershman *et al*. [8] used changepoint analysis to identify 3519 Affymetrix probe-sets that changed their transcript level during 7 hours after feeding. Only 3270 of these probe-sets could be unambiguously identified in *Drosophila *genome version 5.1. For the remainder, at least one probe in the probe-set was similar to more than one genome position using a BLAST algorithm. These 3270 probe-sets correspond to 3171 genes. Oligonucleotides for 3126 out of 3171 genes were represented on the Agilent microarray. Of these, 3048 genes showed significant hybridization and genetic variation in breeding values in females and 3031 of these were identified in males.

To summarize transcript level variation of these approximately 3000 genes, we employed factor analysis. Each gene has 9 biologically independent measurements per sex per probe. Accordingly, we can infer up to 8 axes of transcriptome variation (factors). An individual gene might or might not vary in its expression level along a factor, which is indicated by the magnitude of its loading onto this factor (Additional file 4). In females, the first factor explained 44.35% of the total transcriptome variation with 2296 genes having bigger than 0.4 loading, but only 10 smaller than -0.4 loading. The second factor accounted for 22.17% of the variation contributed by substantial loading of 1306 genes. The third and all subsequent factors explained 12.78% (5.53%, 4.92%, 4.10%, 3.60%, 2.54%) of the transcriptome variation and included 908 (260, 228, 182, 148, 75) genes. In males, likewise, the factors accounted for 49.95, 12.52, 9.95, 7.45, 6.71, 5.47, 4.43, and 3.53 percent of the variation with 2633 (only four of them with negative loading), 874, 666, 438, 371, 275, 194, and 116 genes. The average loading onto the first factor was 0.59 in females and 0.67 in males. The factor loading was very consistent between males and females with most of the same genes loading on the same factors for the two sexes.

We investigated the biological processes and molecular functions of these genes using gene ontology (GO) enrichment analysis. For each of the first three factors in males and females separately, which together accounted for 72.42% and 79.30% of the transcriptome variation respectively, we report all of the GO terms that we found overrepresented in our gene lists using a 0.15 FDR [32] significance cutoff (Additional file 5). For both sexes, the first factor reflected terms related to metabolism, and females were further enriched for terms involved with translation and transport. The second and third factors for both sexes were enriched for terms related to signal transduction and electron transport, respectively.

### (iii) Covariation of transcript levels in dFOXO and TOR branches of InR/TOR network

Out of the 995 dFOXO-affected genes reported by Gershman *et al*. [8], 911 genes were present on the Agilent array. 887 of these showed evidence of variation in females and 872 showed evidence of variation in males. These genes are a subset of their larger list of ~3000 feeding-affected genes described above. We first analyzed whether variation in these dFOXO genes was distinctly directed along the major axes of transcriptome variation. Factor 1 influences the subset of dFOXO-affected genes somewhat more strongly than the rest of the genes in the InR/TOR pathway: the average loading of dFOXO affected genes on the first factor was 0.69 in females and 0.71 in males, while for the rest of the approximately 2000 genes it was somewhat smaller at 0.55 in females and 0.65 in males. dFOXO genes did not consistently co-vary for expression with the other factors. To further assess variation in these genes as a subgroup, we conducted a factor analysis for the dFOXO affected genes separately. The factors from the initial inclusive analysis and the limited analysis of dFOXO targets were largely collinear (data not shown).

Direct targets of dFOXO have been identified using ChIP-chip technology and the binding motifs identified by these assays were found in hundreds of genes [10]. We asked whether or not genotypes with naturally higher levels of dFOXO were more correlated with their dFOXO binding targets. This permits us to test whether or not the dFOXO to downstream target co-variation is explained by the number or quality of binding sites in the proximity of the target gene. We determined the correlation between dFOXO transcript level and the transcript level for the bound gene among the 9 genotypes (Additional file 6). We then compared the correlation of transcript levels of dFOXO and the targets of dFOXO (and their absolute values) to the intensities of binding of the target gene as defined by ChIP-chip analyses. The correlation across all genes was not significant. While the intensity of binding might not be a good predictor of the quality of the downstream gene regulation by dFOXO, we hypothesize that dFOXO transcript level variation is not mechanistically responsible for the variation in the transcript level of its downstream genes among the 9 genotypes. We also made an analogous analysis with the AKT transcript levels. Our rationale is that AKT activity, which is potentially dependent on the AKT transcript level, might be responsible for a varying degree of dFOXO exclusion from the nucleus among genotypes. This would result in varying degrees of the dFOXO binding to the downstream genes. Again, we did not observe this effect (data not shown).

Guertin *et al*. [9] reported approximately 1200 genes that changed their expression level in response to rapamycin treatment and thus are TOR affected genes. However, only a fraction of these genes were reported in the Supplementary Tables available for that manuscript. From this paper, we combined gene lists from Tables S1-S3. Among them, 54 genes had significant transcript level variation in males and females, and 46 were represented in the gene set reported by Gershman *et al*. [8]. Similar to the pattern detected for the dFOXO affected genes, TOR affected genes were tightly coregulated, with the average loading onto the first factor being 0.79 for females and 0.77 for males. Limiting the factor analysis to only the TOR affected genes did not change the direction of axes of transcriptome variation (data not shown), again resembling the pattern detected in the dFOXO branch of InR/TOR pathway.

These factor loadings suggest that the dFOXO affected genes and the TOR affected genes are more tightly covarying than the overall set of feeding-affected genes. To test this hypothesis explicitly we examined the distribution of the pairwise correlation co-efficient in the feeding-affected genes in females and males (average of ρ = 0.35, ρ = 0.38 for females, males, respectively) to the dFOXO subset (ρ = 0.46, ρ = 0.44) and the TOR subgroup (ρ = 0.78, ρ = 0.77). Random samples of the feeding-affected gene list of the size of the dFOXO and TOR subgroups were taken 1000 times and the average pairwise correlation calculated. We conclude that at *P *< 0.001 dFOXO affected genes [8] and TOR affected genes [9] are more tightly co-varying sub-groups of a larger group of 3500 feeding-affected genes. However, the major axes of transcript level variation in these two subsets of genes do not seem to differ from those of the larger group of feeding-affected genes.

### (iv) Partial regression analyses of multiply connected genes

For the well-established connections of the InR/TOR pathway, we have extended our analyses beyond pairwise comparisons to include multiple connections via partial correlation analysis following Neto et. al. [33]. We analyzed all the multiply connected genes in the pathway, but in many cases partial regression analyses did not add extra inferences to the simple pairwise correlations. Instances where it did are reported in Table 1. Here, we briefly summarize them. In the pairwise models, TOR is strongly associated with TSC1, *slimfast *(*slif) *and *myc *but not Rheb. In further examination of the pairwise associations, we find that Rheb is not associated with *slif *or *myc *and in contrast to the visualization of the pathway (Figure 1) is significantly correlated with TSC1, *chico *and *eIF4E*. Considering a larger model and partial correlations, if *myc *is the final product of the pathway (the dependent variable), then we can construct a model and examine the sequential contribution of *TOR*, AKT, *Rheb*, *TSC1 *and *slif*. As expected, the effect of TOR is sufficient to predict *myc *and in this larger model the sequential effects of the remaining genes are not statistically significant. This fully validated the strong link between TOR and *myc*. If instead TOR we examine a model where TOR is the dependent variable and *Rheb*, TSC1, and *slif *are considered sequentially, TSC1 is still significant after considering the effects of *Rheb*, and *slif *is still significant after accounting for the effects of *Rheb *and TSC1. This suggests that the proposed pathway (Figure 1) is not complete and that there is likely (in non-stress conditions) to be a direct link between *slif *and TOR, and between TSC1 and TOR as well. To see if the link between TSC1 and TOR was mediated by *AKT*, a model with *AKT *upstream of TSC1 and *slif *was fit. After accounting for the variation in *AKT*, TSC1 was still significant, as was *slif*, indicating that AKT is unlikely to explain the feedback loop completely. In contrast, when *S6K *was placed downstream of AKT, S6K was no longer significantly associated with TOR, indicating that as previously reported the effect of S6K is mediated by AKT. We consider the above logical conjectures to be somewhat preliminary for two reasons. First, when we see a significant partial correlation between the genes, it might be caused not by their direct interactions, but rather by the cumulative effects of variation elsewhere in the network that we have not accounted for. Second, when we do not detect a significant partial correlation, it might be due to limited genetic variation within our 9 breeding values and thus to insufficient power.

**Table 1:** Linear models with dependent effects given to the left of the equals sign and independent effects to the right of the equals sign.

Model	P-values* in the order given by the model
Myc = TOR + AKT + Rheb + TSC1 + slif + e	0.0064, 0.0555, 0.0905, 0.7840, 0.3521

TOR = Rheb + TSC1 + slif + e	0.0002, 0.0001, 0.0099

TOR = Rheb + AKT1+TSC1+ slif +e	0.0001, 0.0001, 0.002, 0.0003

TOR = AKT + Pdk1+ S6K+ e	0.0017, 0.5166, 0.0724

* P-values for the tests of the sequential sums of squares (type I tests) in females.

### (v) Genotype-to-phenotype map exhibits some effects expected from the analyses of major effect mutations

Major effect mutations in the InR/TOR pathway affect lifespan, oxidative stress resistance, body size and other phenotypes (see Introduction for details). We analyzed whether the mean trait values of these phenotypes among the 72 genotypes in males and females genetically co-vary with transcript levels of individual genes in InR/TOR pathway. Detailed analyses of the phenotypic data are reported elsewhere (Wayne et al. [16] and Harshman, personal communication) and here we only present important highlights. We have observed several effects expected from analyses of large-effect mutations (Table 2). For instance, knock-outs of the dFOXO gene are known to have improved oxidative stress resistance [28]. We therefore hypothesized that genotypes with weaker dFOXO expression would likewise show stronger oxidative resistance. This expectation holds true in both sexes. Likewise, other genes exhibit expected genetic co-variations with multiple phenotypes, though none of these effects is significant after multiple testing correction. Furthermore, it remains unclear which genes in this signal transduction cascade or in its upstream regulators have causal effects on these phenotypes because the network appears strongly co-regulated and lacking finer structure. We conclude that while the phenotypic effects of these network perturbations reflect *a priori *expectations, it is difficult, perhaps impossible, to identify causal genes responsible for the phenotypic alterations without additional information.

**Table 2:** Correlations of individual genes with each of five phenotypes in females.

Gene	Desiccation resistance	Oxidative stress	Starvation	Longevity	Development time	Bodysize
Akt1	-0.34	*-0.75*,* 0.02*	-0.43	-0.46	-0.01	-0.37

InR	0.10	-0.63	-0.22	-0.11	0.25	0.24

Lk6	-0.30	*-0.81*,* 0.005*	-0.30	-0.47	-0.06	-0.13

Pi3K	-0.31	*-0.76*,* 0.01*	-0.44	-0.58	0.07	-0.01

PDK1	-0.41	*-0.67*, *0.04*	-0.27	-0.33	-0.01	-0.19

PTEN	-0.29	*-0.72*, *0.03*	-0.50	-0.43	0.02	-0.59

Rheb	-0.18	-0.44	0.11	-0.24	0.02	-0.44

S6K	-0.29	-0.43	-0.24	0.28	0.32	0.03

4EBP	0.27	-0.04	0.09	-0.47	*-0.80*, *0.008*	*-0.71*, *0.03*

Tor	-0.32	*-0.88*, *0.0008*	-0.49	-0.53	0.05	-0.01

Tsc1	-0.33	*-0.69*, *0.04*	-0.24	-0.39	0.07	-0.20

chico	-0.18	-0.44	0.14	-0.17	0.01	-0.24

Myc	-0.15	-0.60	-0.17	-0.35	-0.06	0.31

eIF4E	-0.43	-0.53	-0.21	-0.45	-0.02	-0.46

FOXO	-0.42	***-0.93*****,*****6.2******×******10***^***-5***^	*-0.79*, *0.008*	-0.41	0.23	0.04

Tsc2	-0.42	*-0.89*, *0.0005*	-0.45	-0.55	0.03	0.02

Slif	-0.06	*-0.80*, *0.008*	-0.37	-0.48	-0.15	-0.47

Male values are similar and can be found in Additional file 3. If the correlation was significant, then the correlation coefficient is italicized and the P-value is given. P-values that remain significant at the 0.05 level following Bonferroni correction are bolded.

In (ii) and (iii), we summarized overall transcript level variation using factor analysis. We asked whether or not these major axes of transcriptome variation are better predictors of phenotypic variation than the upstream genes of the InR/TOR pathway by correlating the values for these factors with the phenotypes among genotypes (Table 3). While we have detected several suggestive patterns summarized in Additional file 3, none of them stood out after correction for multiple testing.

**Table 3:** Correlations of factors with each of five phenotypes in females.

Factor	Desiccation resistance	Oxidative stress	Starvation	Longevity	Development time	Bodysize
Factor 1	-0.32	-0.62	-0.15	-0.34	-0.07	-0.29

Factor 2	0.12	0.31	-0.25	-0.11	-0.07	0.56

Factor 3	0.12	0.64	0.62	0.35	-0.30	0.24

Factor 4	0.02	0.01	-0.16	0.29	-0.08	-0.27

Factor 5	0.23	-0.04	0.44	0.46	0.24	0.05

Factor 6	0.41	0.03	0.02	-0.51	*-0.82*, *0.005*	-0.49

Factor 7	*0.81*, *0.005*	0.31	0.53	0.42	0.02	-0.48

Factor 8	-0.02	0.08	0.17	0.15	-0.41	-0.08

Male values are similar and can be found in Additional file 3. If the correlation was significant, then the correlation coefficient is italicized and the P-value is given for that correlation. No P-values remained significant after Bonferroni correction.

## Discussion

A common method of reconstructing and characterizing gene regulatory networks is to individually analyze the transcriptional profiles of a collection of single gene knockouts and infer regulatory relationships based on gene expression changes [for recent applications and a review, see [34-37]]. While these approaches have yielded important information regarding gene regulatory networks, the methods are limited given the cost and labor required to determine the expression profile for a knockout in every gene. Furthermore, analyzing gene knockouts does not allow for an assessment of smaller effect perturbations in genes, which may be a more common type of genetic variation in natural populations. One way around these limitations is to examine and analyze gene expression from many individual genotypes that each contains multiple mutations and to study the covariance in phenotypes and multiple gene expression levels to hypothesize which of the gene expression changes might cause phenotypic deviations [38-41]. If successful, this would contribute to elucidating components of the genotype-to-phenotype map. It might also greatly aid our understanding of the genetic basis of human disease, given that most human disease is caused by small effect mutations in many genes [42-44].

If most genetic regulatory networks are sparse, meaning that there is not a substantial overlap in the genes regulated within different networks [45], it should be possible to analyze a relatively small number of genotypes and use mathematical models to infer regulatory relationships between many genes. Major effect mutations do affect some downstream genes more strongly than others, thus establishing main regulatory connections. However, these strong effects also mask numerous weaker connections. Indeed, a major effect mutation typically disturbs hundreds or thousands of the genes, albeit to different degrees [34-37], as revealed by whole genome analyses of transcriptome alterations. With more powerful experimental and statistical approaches, these effects can likely be detected on many thousands of genes, or even onto all of the transcriptome. While with single major effect mutation per genotype, the main regulatory connections are easy to recognize, would this also hold true for natural genetic variation? More specifically, are genetic networks sparse enough for naturally segregating smaller-effect mutations to reveal individual network connections? From the analyses of transcriptome data presented here, it appears that the collective effects of numerous natural mutations might override a sparse nature of major effect mutations and dominate the collective properties of molecular networks.

In unrelated wild type flies, every genotype contains numerous sequence alterations. With approximately 1/100 bases different between two random unrelated flies, the total number of whole-genome differences is in the millions. How many of these differences represent regulatory mutations is not known, but some estimates may be offered as follows. Genissel *et al*. [46] investigated whole genome expression from an oligonucleotide microarray in two extensively studied genotypes of *Drosophila melanogaster*, Ore and 2b3, and six recombinant inbred lines derived from these parents. Approximately 10% of the transcriptome was differentially regulated among the lines. Regulatory effects in *cis *(regulatory mutations in the gene locus itself or nearby) appeared present in up to 1218 genes. 123 genes were affected by *trans *mutations, but the vast majority of *trans *effects probably remained undetected because there was not enough consistency among different analytical procedures. Wang *et al*. [47] assayed a full set of chromosome substitution lines between the two behavioral races of *D. melanogaster *Z and M. Only about 3% of the genes with an expression difference between races were purely *cis *regulated, while transcript levels of 80% of the genes were controlled by at least two different chromosomes. From these two studies, we can conclude that hundreds to thousands of genes in each genotype contain regulatory alterations. Therefore, regulatory mutations likely affect hundreds of networks in each genotype, and quite a few of them many times. These mutations in turn affect thousands or tens of thousands of downstream targets. If we now recall that our data set combines the analysis of variation over 9 genotypes, it becomes clear that the patterns of covariance we observe represents not individual gene-to-gene connections, but rather system-wide effects of massive genetic variation onto transcriptome architecture.

Why, then, have some analyses [see [38] for summary, [48]] been able to reconstruct quite a few network connections? We believe that there are two main reasons for this, both stemming from the fact that typical analyses have been based on Recombinant Inbred Lines (RILs). First, researchers mapped factors affecting transcription of focal genes to *cis *(position of the focal gene) and other regions of the genome (*trans*). With approximately a hundred RILs, only the strongest effects could have been unambiguously mapped, especially after corrections for the number of tested genes. Most regulatory connections must have been missed, although their composite effects might be overwhelming. Contrary to this supposition, the factors mapped typically accounted for an appreciable portion of the focal gene's transcript variation. We argue, though, that the Beavis effect, rather than the true Mendelian nature of the mapped factors, is a likely reason for such conclusions, meaning that when the power of QTL mapping is low, the effects of *detected *QTLs are strongly overestimated [49]. The requirement to correct for multiple tested genes in the whole transcriptome eQTL mapping makes the power very low even in the best eQTL mappings. Accordingly, these experiments are likely to i) detect only the strongest effects and ii) overestimate their effects. This should result in a substantially oversimplified picture of genetic network connectedness. Additionally, a set of RILs is typically built from just two parental genotypes. We argue that both alleles sampled per gene (one from the first and another from the second parent) are likely to be functionally equivalent. Whenever the genetic network is connected through the gene in which there is no variation in the mapping RIL population, the network will appear 'broken' at this gene. This will contribute to apparent 'resolution' of the network connections. Overall, we feel that the eQTL based networks are likely to be oversimplified. We believe that higher power and larger genetic base network analyses will recover, in the future, much more connected and complex genetic networks.

## Conclusion

We report one of the first attempts to use a population with a broad genetic basis to decipher the nature of genetic variation in the transcriptome and to link it with phenotypic variation. While the genetic architecture of transcriptome variation appears very complex, the use of prior information about the InR/TOR signaling cascade allows several interesting inferences. First, the transcript levels of the genes connected within the network appear coordinated. Second, transcript level variation is reflected in phenotypic variation in expected ways predicted from mutational analyses. Third, new connections are proposed that will help to focus further molecular analyses of InR/TOR network.

## Methods

### Genetic material and expression measurements

The microarray data acquisition and analysis were described by Wayne *et al*. [16]. Briefly, nine isogenic lines of *D. melanogaster *used as parents were originally captured in an orchard in Winters, CA, and subjected to > 20 generations of full sibling inbreeding. Lines were crossed in a full diallel design with reciprocals, but without homozygous parents (72 F1 progeny). RNA was extracted from 20 whole 3-day post eclosion flies, snap-frozen in liquid nitrogen using Trizol reagent (Invitrogen, Carlsbad, CA). The chip was synthesized on an Agilent platform (, AMADID 012798; 3). Hybridizations were performed with males and females of the same genotype, labeled in contrasting dyes, hybridized to the same chip. We analyzed two independent biological replicates for each genotype and sex combination. Intensity values were normalized using the natural log transformation. The chip design contained 503 negative control sequences designed from human sequences with similar GC content to Drosophila, but with no known sequence homology. For each slide and dye combination the 90% value of the negative controls was used as the detection threshold. For a particular probe to be used both replicates needed to be detected. Probes that were not detected in at least one genotype were eliminated from further consideration for that sex. Additionally, the probe needed to show significant variation for expression for either genotype or sex to be considered further. For the probes that were detected, those corresponding to the list of genes identified as part of the dFOXO regulatory cascade (n = 1301 probes) were identified as described above. We found 1281 probes representing 887 genes in females and 1262 probes representing 872 genes in males.

### Factor analytical techniques

Initial factor analysis was conducted by determining the eigenvalues for the matrix of genes, first for the feeding-affected genes and second for the subgroup of genes in the dFOXO pathway. Principle components factor analysis was used to estimate factor loads for 8 factors for both males and females. A gene was considered to contribute significantly to the factor if the estimated loading value had an absolute value of 0.4 or higher.

### Gene Ontology Analysis

Genes with load scores greater than 0.4 or less than -0.4 were used to define gene lists for over-representation analysis of gene ontology (GO) terms implemented in DAVID (found at [50]). The P-value reported is produced from a modified Fisher's Exact Test called the EASE Score [51] computed in DAVID.

### Phenotypic analyses

For the stress assays, mortality was recorded multiple times each day and for the life span assay mortality was recorded at three-day intervals. Oxidative stress survival was determined for flies held on methyl viologen as an oxidant. Starvation survival was measured in the absence of food at high humidity and with access to water. Desiccation survival was measured in a container with a desiccant. Life span was determined in small population cages within which flies had continuous access to food. Development time was the time between placing eggs on Drosophila food and the time of adult emergence from pupae (L. Harshman, unpublished data). Ovariole number per ovary was scored from three females from each of two replicate vials. Body size was estimated by measuring thorax length on ten males and ten females from each of two replicate vials [52].

### Network analysis

For three expression profiles, G_1_, G_2_, and G_3_, consider the model where G_1 _affects G_2_, and G_2 _affects G_3_. In this case, G_2 _= α + β_1 _G_1_+ ε and G_3 _= α + β_2 _G_2 _+ ε, thus, G_3 _= α + β_2 _G_2 _+ β_1_G_1 _+ ε. In the first two models, we expect the effects β_1 _and β_2 _to be significant, while in the last model the partial regression coefficient of β_1_should not be significant since the effect of G_2 _has been already accounted for in the model [33,53]. Using this logic, the order of the pathway in Figure 1 can be tested. For example, to test the hypothesis that *Rheb *only affects *myc *via TOR, the model *myc *= α + β TOR + β *Rheb *+ e is fit and the partial regression coefficient for *Rheb *is examined. If the hypothesis is false, then β *Rheb *will be different from zero. In this way, a series of models examining the relationships proposed by the known pathway can be tested.

## Authors' contributions

SVN co-analyzed the data with AP, JAB and LMM, and wrote the first draft of the manuscript. JAB also assisted in preparing the manuscript. LMM contributed to the remainder of the data analyses and assisted in preparing the manuscript. MLW and LH provided phenotypic data and commented on the manuscript.

## Supplementary Material

Additional File 1 **Male breeding values for transcript levels in the 9 parental genotypes.** Click here for file

Additional File 2 **Female breeding values for transcript levels in the 9 parental genotypes.** Click here for file

Additional File 3 **All pairwise correlations between the transcript levels of the genes in Figure**1, **between transcript levels and phenotypes, or between factors and phenotypes for females (worksheet 1) and males (worksheet 2).** Click here for file

Additional File 4 **The loading values of each gene onto each of the eight possible factors in males and females and a set of indicator variables to indicate whether a gene was considered to load onto a factor as well as a set of indicator variables for genes that load on the same factor in both sexes.** Click here for file

Additional File 5 **Lists all of the significantly enriched (*P *< 0.01, FDR < 0.15) GO biological process (BP) and molecular function (MF) terms associated with factors 1, 2 and 3 for females (F) and males (M).** Click here for file

Additional File 6 **Pairwise correlations between AKT or dFOXO and genes reported by ChIP-chip**[10]**to bind todFOXO**. Click here for file
