# Supplementary material for: Natural genetic variation in transcriptome reflects network structure inferred with major effect mutations: insulin/TOR and associated phenotypes in Drosophila melanogaster
Source: BMC Genomics. 2009 Mar 24;10:124. doi: 10.1186/1471-2164-10-124 (PMC2674066; doi:10.1186/1471-2164-10-124)
Supplement: Additional File 5 — Lists all of the significantly enriched (P < 0.01, FDR < 0.15) GO biological process (BP) and molecular function (MF) terms associated with factors 1, 2 and 3 for females (F) and males (M). [file 1471-2164-10-124-S5.doc]

Additional File 5. Significantly enriched (*P* < 0.01, FDR < 0.15) GO biological process (BP) and molecular function (MF) terms associated with factors 1, 2 and 3 for females (F) and males (M).

| Sex | Factor | GO Category | Term | P | FDR |
| --- | --- | --- | --- | --- | --- |
| F | 1 | BP | GO:0044238 primary metabolic process | 0.0000 | 0.0000 |
| F | 1 | BP | GO:0044237 cellular metabolic process | 0.0000 | 0.0000 |
| F | 1 | BP | GO:0043170 macromolecule metabolic process | 0.0000 | 0.0000 |
| F | 1 | BP | GO:0044267 cellular protein metabolic process | 0.0000 | 0.0000 |
| F | 1 | BP | GO:0006139 nucleobase, nucleoside, nucleotide & nucleic acid metabolic process | 0.0000 | 0.0000 |
| F | 1 | BP | GO:0019538 protein metabolic process | 0.0000 | 0.0000 |
| F | 1 | BP | GO:0044260 cellular macromolecule metabolic process | 0.0000 | 0.0000 |
| F | 1 | BP | GO:0022607 cellular component assembly | 0.0000 | 0.0000 |
| F | 1 | BP | GO:0010467 gene expression | 0.0000 | 0.0001 |
| F | 1 | BP | GO:0009059 macromolecule biosynthetic process | 0.0000 | 0.0001 |
| F | 1 | BP | GO:0006412 translation | 0.0000 | 0.0001 |
| F | 1 | BP | GO:0009058 biosynthetic process | 0.0000 | 0.0001 |
| F | 1 | BP | GO:0022402 cell cycle process | 0.0000 | 0.0002 |
| F | 1 | BP | GO:0043283 biopolymer metabolic process | 0.0000 | 0.0002 |
| F | 1 | BP | GO:0044249 cellular biosynthetic process | 0.0000 | 0.0003 |
| F | 1 | BP | GO:0022403 cell cycle phase | 0.0000 | 0.0008 |
| F | 1 | BP | GO:0022613 ribonucleoprotein complex biogenesis & assembly | 0.0001 | 0.0012 |
| F | 1 | BP | GO:0007049 cell cycle | 0.0001 | 0.0016 |
| F | 1 | BP | GO:0006457 protein folding | 0.0001 | 0.0019 |
| F | 1 | BP | GO:0033036 macromolecule localization | 0.0001 | 0.0021 |
| F | 1 | BP | GO:0000279 M phase | 0.0001 | 0.0025 |
| F | 1 | BP | GO:0008104 protein localization | 0.0002 | 0.0038 |
| F | 1 | BP | GO:0065003 macromolecular complex assembly | 0.0003 | 0.0055 |
| F | 1 | BP | GO:0015031 protein transport | 0.0003 | 0.0061 |
| F | 1 | BP | GO:0000278 mitotic cell cycle | 0.0004 | 0.0078 |
| F | 1 | BP | GO:0045184 establishment of protein localization | 0.0006 | 0.0104 |
| F | 1 | BP | GO:0016043 cellular component organization & biogenesis | 0.0006 | 0.0114 |
| F | 1 | BP | GO:0006259 DNA metabolic process | 0.0011 | 0.0186 |
| F | 1 | BP | GO:0006996 organelle organization & biogenesis | 0.0011 | 0.0196 |
| F | 1 | BP | GO:0000087 M phase of mitotic cell cycle | 0.0011 | 0.0197 |
| F | 1 | BP | GO:0007067 mitosis | 0.0011 | 0.0197 |
| F | 1 | BP | GO:0046907 intracellular transport | 0.0011 | 0.0200 |
| F | 1 | BP | GO:0006886 intracellular protein transport | 0.0023 | 0.0396 |
| F | 1 | BP | GO:0016070 RNA metabolic process | 0.0023 | 0.0407 |
| F | 1 | BP | GO:0006974 response to DNA damage stimulus | 0.0029 | 0.0498 |
| F | 1 | BP | GO:0006396 RNA processing | 0.0030 | 0.0525 |
| F | 1 | BP | GO:0009719 response to endogenous stimulus | 0.0036 | 0.0624 |
| F | 1 | BP | GO:0006512 ubiquitin cycle | 0.0040 | 0.0679 |
| F | 1 | BP | GO:0006281 DNA repair | 0.0056 | 0.0945 |
| F | 1 | BP | GO:0006605 protein targeting | 0.0056 | 0.0951 |
| F | 1 | BP | GO:0006950 response to stress | 0.0063 | 0.1061 |
| F | 1 | BP | GO:0051641 cellular localization | 0.0066 | 0.1100 |
| F | 1 | BP | GO:0022618 protein-RNA complex assembly | 0.0067 | 0.1117 |
| F | 1 | MF | GO:0003676 nucleic acid binding | 0.0000 | 0.0002 |
| F | 1 | MF | GO:0005515 protein binding | 0.0001 | 0.0019 |
| F | 1 | MF | GO:0019001 guanyl nucleotide binding | 0.0003 | 0.0049 |
| F | 1 | MF | GO:0032561 guanyl ribonucleotide binding | 0.0004 | 0.0060 |
| F | 1 | MF | GO:0003723 RNA binding | 0.0004 | 0.0067 |
| F | 1 | MF | GO:0005525 GTP binding | 0.0004 | 0.0073 |
| F | 1 | MF | GO:0005488 binding | 0.0007 | 0.0113 |
| F | 1 | MF | GO:0000166 nucleotide binding | 0.0009 | 0.0147 |
| F | 1 | MF | GO:0008135 translation factor activity, nucleic acid binding | 0.0011 | 0.0186 |
| F | 1 | MF | GO:0003729 mRNA binding | 0.0014 | 0.0235 |
| F | 1 | MF | GO:0016874 ligase activity | 0.0017 | 0.0277 |
| F | 1 | MF | GO:0032555 purine ribonucleotide binding | 0.0035 | 0.0559 |
| F | 1 | MF | GO:0032553 ribonucleotide binding | 0.0035 | 0.0559 |
| F | 1 | MF | GO:0008565 protein transporter activity | 0.0037 | 0.0595 |
| F | 1 | MF | GO:0016462 pyrophosphatase activity | 0.0040 | 0.0635 |
| F | 1 | MF | GO:0045182 translation regulator activity | 0.0043 | 0.0677 |
| F | 1 | MF | GO:0017076 purine nucleotide binding | 0.0048 | 0.0761 |
| F | 1 | MF | GO:0017111 nucleoside-triphosphatase activity | 0.0050 | 0.0790 |
| F | 1 | MF | GO:0016818 hydrolase activity, acting on acid anhydrides, in phosphorus-containing anhydrides | 0.0051 | 0.0802 |
| F | 1 | MF | GO:0016817 hydrolase activity, acting on acid anhydrides | 0.0051 | 0.0802 |
| F | 1 | MF | GO:0051082 unfolded protein binding | 0.0053 | 0.0830 |
| F | 1 | MF | GO:0003824 catalytic activity | 0.0056 | 0.0888 |
| F | 1 | MF | GO:0003924 GTPase activity | 0.0074 | 0.1154 |
| F | 2 | BP | GO:0007154 cell communication | 0.0000 | 0.0000 |
| F | 2 | BP | GO:0007417 central nervous system development | 0.0000 | 0.0000 |
| F | 2 | BP | GO:0007165 signal transduction | 0.0000 | 0.0001 |
| F | 2 | BP | GO:0007626 locomotory behavior | 0.0000 | 0.0003 |
| F | 2 | BP | GO:0050877 neurological system process | 0.0000 | 0.0006 |
| F | 2 | BP | GO:0003008 system process | 0.0000 | 0.0006 |
| F | 2 | BP | GO:0007610 behavior | 0.0001 | 0.0011 |
| F | 2 | BP | GO:0019226 transmission of nerve impulse | 0.0002 | 0.0031 |
| F | 2 | BP | GO:0007267 cell-cell signaling | 0.0003 | 0.0051 |
| F | 2 | BP | GO:0032501 multicellular organismal process | 0.0003 | 0.0051 |
| F | 2 | BP | GO:0006811 ion transport | 0.0003 | 0.0052 |
| F | 2 | BP | GO:0051056 regulation of small GTPase mediated signal transduction | 0.0006 | 0.0112 |
| F | 2 | BP | GO:0007265 Ras protein signal transduction | 0.0012 | 0.0204 |
| F | 2 | BP | GO:0009966 regulation of signal transduction | 0.0012 | 0.0211 |
| F | 2 | BP | GO:0007268 synaptic transmission | 0.0013 | 0.0221 |
| F | 2 | BP | GO:0046578 regulation of Ras protein signal transduction | 0.0013 | 0.0222 |
| F | 2 | BP | GO:0007399 nervous system development | 0.0013 | 0.0232 |
| F | 2 | BP | GO:0043473 pigmentation | 0.0017 | 0.0302 |
| F | 2 | BP | GO:0007517 muscle development | 0.0018 | 0.0306 |
| F | 2 | BP | GO:0007155 cell adhesion | 0.0027 | 0.0462 |
| F | 2 | BP | GO:0022610 biological adhesion | 0.0027 | 0.0462 |
| F | 2 | BP | GO:0032990 cell part morphogenesis | 0.0028 | 0.0486 |
| F | 2 | BP | GO:0030030 cell projection organization & biogenesis | 0.0028 | 0.0486 |
| F | 2 | BP | GO:0048858 cell projection morphogenesis | 0.0028 | 0.0486 |
| F | 2 | BP | GO:0050789 regulation of biological process | 0.0029 | 0.0509 |
| F | 2 | BP | GO:0048066 pigmentation during development | 0.0034 | 0.0582 |
| F | 2 | BP | GO:0007242 intracellular signaling cascade | 0.0037 | 0.0637 |
| F | 2 | BP | GO:0030001 metal ion transport | 0.0045 | 0.0774 |
| F | 2 | BP | GO:0032502 developmental process | 0.0048 | 0.0825 |
| F | 2 | BP | GO:0007264 small GTPase mediated signal transduction | 0.0057 | 0.0969 |
| F | 2 | BP | GO:0050793 regulation of developmental process | 0.0059 | 0.0990 |
| F | 2 | BP | GO:0048513 organ development | 0.0063 | 0.1056 |
| F | 2 | BP | GO:0016331 morphogenesis of embryonic epithelium | 0.0064 | 0.1081 |
| F | 2 | BP | GO:0048856 anatomical structure development | 0.0066 | 0.1109 |
| F | 2 | BP | GO:0065007 biological regulation | 0.0069 | 0.1146 |
| F | 2 | BP | GO:0000904 cellular morphogenesis during differentiation | 0.0077 | 0.1280 |
| F | 2 | BP | GO:0007166 cell surface receptor linked signal transduction | 0.0090 | 0.1473 |
| F | 2 | BP | GO:0030154 cell differentiation | 0.0090 | 0.1482 |
| F | 2 | MF | GO:0004871 signal transducer activity | 0.0000 | 0.0000 |
| F | 2 | MF | GO:0060089 molecular transducer activity | 0.0000 | 0.0000 |
| F | 2 | MF | GO:0004872 receptor activity | 0.0000 | 0.0000 |
| F | 2 | MF | GO:0004888 transmembrane receptor activity | 0.0000 | 0.0000 |
| F | 2 | MF | GO:0022836 gated channel activity | 0.0000 | 0.0001 |
| F | 2 | MF | GO:0005216 ion channel activity | 0.0000 | 0.0002 |
| F | 2 | MF | GO:0022838 substrate specific channel activity | 0.0000 | 0.0004 |
| F | 2 | MF | GO:0005261 cation channel activity | 0.0000 | 0.0007 |
| F | 2 | MF | GO:0022834 ligand-gated channel activity | 0.0001 | 0.0016 |
| F | 2 | MF | GO:0015276 ligand-gated ion channel activity | 0.0001 | 0.0016 |
| F | 2 | MF | GO:0015075 ion transmembrane transporter activity | 0.0004 | 0.0067 |
| F | 2 | MF | GO:0046873 metal ion transmembrane transporter activity | 0.0006 | 0.0103 |
| F | 2 | MF | GO:0030695 GTPase regulator activity | 0.0007 | 0.0107 |
| F | 2 | MF | GO:0005230 extracellular ligand-gated ion channel activity | 0.0011 | 0.0174 |
| F | 2 | MF | GO:0022803 passive transmembrane transporter activity | 0.0013 | 0.0218 |
| F | 2 | MF | GO:0015267 channel activity | 0.0013 | 0.0218 |
| F | 2 | MF | GO:0003779 actin binding | 0.0019 | 0.0307 |
| F | 2 | MF | GO:0005083 small GTPase regulator activity | 0.0021 | 0.0333 |
| F | 2 | MF | GO:0005085 guanyl-nucleotide exchange factor activity | 0.0036 | 0.0577 |
| F | 2 | MF | GO:0022891 substrate-specific transmembrane transporter activity | 0.0038 | 0.0606 |
| F | 2 | MF | GO:0008324 cation transmembrane transporter activity | 0.0071 | 0.1101 |
| F | 2 | MF | GO:0003704 specific RNA polymerase II transcription factor activity | 0.0081 | 0.1253 |
| F | 2 | MF | GO:0015294 solute:cation symporter activity | 0.0085 | 0.1316 |
| F | 3 | BP | GO:0006091 generation of precursor metabolites & energy | 0.0000 | 0.0000 |
| F | 3 | BP | GO:0006119 oxidative phosphorylation | 0.0000 | 0.0000 |
| F | 3 | BP | GO:0006118 electron transport | 0.0000 | 0.0000 |
| F | 3 | BP | GO:0042775 organelle ATP synthesis coupled electron transport | 0.0000 | 0.0000 |
| F | 3 | BP | GO:0042773 ATP synthesis coupled electron transport | 0.0000 | 0.0000 |
| F | 3 | BP | GO:0006120 mitochondrial electron transport, NADH to ubiquinone | 0.0000 | 0.0000 |
| F | 3 | BP | GO:0044262 cellular carbohydrate metabolic process | 0.0001 | 0.0020 |
| F | 3 | BP | GO:0005975 carbohydrate metabolic process | 0.0001 | 0.0022 |
| F | 3 | BP | GO:0009144 purine nucleoside triphosphate metabolic process | 0.0030 | 0.0526 |
| F | 3 | BP | GO:0009205 purine ribonucleoside triphosphate metabolic process | 0.0030 | 0.0526 |
| F | 3 | BP | GO:0009199 ribonucleoside triphosphate metabolic process | 0.0046 | 0.0779 |
| F | 3 | BP | GO:0044270 nitrogen compound catabolic process | 0.0066 | 0.1109 |
| F | 3 | BP | GO:0009310 amine catabolic process | 0.0066 | 0.1109 |
| F | 3 | BP | GO:0009259 ribonucleotide metabolic process | 0.0077 | 0.1275 |
| F | 3 | MF | GO:0016491 oxidoreductase activity | 0.0000 | 0.0000 |
| F | 3 | MF | GO:0009055 electron carrier activity | 0.0000 | 0.0000 |
| F | 3 | MF | GO:0003954 NADH dehydrogenase activity | 0.0000 | 0.0000 |
| F | 3 | MF | GO:0016651 oxidoreductase activity, acting on NADH or NADPH | 0.0000 | 0.0000 |
| F | 3 | MF | GO:0004252 serine-type endopeptidase activity | 0.0000 | 0.0002 |
| F | 3 | MF | GO:0016655 oxidoreductase activity, acting on NADH or NADPH, quinone or similar compound as acceptor | 0.0000 | 0.0002 |
| F | 3 | MF | GO:0050136 NADH dehydrogenase (quinone) activity | 0.0000 | 0.0002 |
| F | 3 | MF | GO:0008137 NADH dehydrogenase (ubiquinone) activity | 0.0000 | 0.0002 |
| F | 3 | MF | GO:0008236 serine-type peptidase activity | 0.0000 | 0.0005 |
| F | 3 | MF | GO:0017171 serine hydrolase activity | 0.0000 | 0.0005 |
| F | 3 | MF | GO:0020037 heme binding | 0.0001 | 0.0014 |
| F | 3 | MF | GO:0046906 tetrapyrrole binding | 0.0001 | 0.0014 |
| F | 3 | MF | GO:0005506 iron ion binding | 0.0001 | 0.0024 |
| F | 3 | MF | GO:0015078 hydrogen ion transmembrane transporter activity | 0.0002 | 0.0027 |
| F | 3 | MF | GO:0015077 monovalent inorganic cation transmembrane transporter activity | 0.0002 | 0.0027 |
| F | 3 | MF | GO:0022890 inorganic cation transmembrane transporter activity | 0.0003 | 0.0045 |
| F | 3 | MF | GO:0015075 ion transmembrane transporter activity | 0.0020 | 0.0325 |
| F | 3 | MF | GO:0003824 catalytic activity | 0.0023 | 0.0364 |
| F | 3 | MF | GO:0008324 cation transmembrane transporter activity | 0.0087 | 0.1336 |
| F | 3 | MF | GO:0004497 monooxygenase activity | 0.0093 | 0.1423 |
| M | 1 | BP | GO:0006139 nucleobase, nucleoside, nucleotide & nucleic acid metabolic process | 0.0000 | 0.0001 |
| M | 1 | BP | GO:0016070 RNA metabolic process | 0.0000 | 0.0003 |
| M | 1 | BP | GO:0009987 cellular process | 0.0000 | 0.0003 |
| M | 1 | BP | GO:0043283 biopolymer metabolic process | 0.0000 | 0.0004 |
| M | 1 | BP | GO:0010467 gene expression | 0.0009 | 0.0153 |
| M | 1 | BP | GO:0016071 mRNA metabolic process | 0.0009 | 0.0161 |
| M | 1 | BP | GO:0006397 mRNA processing | 0.0014 | 0.0241 |
| M | 1 | BP | GO:0050789 regulation of biological process | 0.0017 | 0.0300 |
| M | 1 | BP | GO:0044238 primary metabolic process | 0.0025 | 0.0428 |
| M | 1 | BP | GO:0016043 cellular component organization & biogenesis | 0.0025 | 0.0439 |
| M | 1 | BP | GO:0006396 RNA processing | 0.0027 | 0.0460 |
| M | 1 | BP | GO:0050794 regulation of cellular process | 0.0033 | 0.0570 |
| M | 1 | BP | GO:0000278 mitotic cell cycle | 0.0034 | 0.0579 |
| M | 1 | BP | GO:0065007 biological regulation | 0.0049 | 0.0832 |
| M | 1 | BP | GO:0043170 macromolecule metabolic process | 0.0055 | 0.0939 |
| M | 1 | BP | GO:0044237 cellular metabolic process | 0.0077 | 0.1286 |
| M | 1 | MF | GO:0003676 nucleic acid binding | 0.0000 | 0.0007 |
| M | 1 | MF | GO:0005515 protein binding | 0.0002 | 0.0029 |
| M | 1 | MF | GO:0003729 mRNA binding | 0.0007 | 0.0108 |
| M | 1 | MF | GO:0003723 RNA binding | 0.0013 | 0.0212 |
| M | 2 | BP | GO:0007417 central nervous system development | 0.0053 | 0.0902 |
| M | 2 | BP | GO:0007200 G-protein signaling, coupled to IP3 second messenger (phospholipase C activating) | 0.0070 | 0.1166 |
| M | 2 | BP | GO:0048015 phosphoinositide-mediated signaling | 0.0070 | 0.1166 |
| M | 2 | BP | GO:0046578 regulation of Ras protein signal transduction | 0.0072 | 0.1197 |
| M | 2 | BP | GO:0007265 Ras protein signal transduction | 0.0075 | 0.1244 |
| M | 2 | MF | GO:0030695 GTPase regulator activity | 0.0015 | 0.0242 |
| M | 3 | BP | GO:0005975 carbohydrate metabolic process | 0.0000 | 0.0000 |
| M | 3 | BP | GO:0006091 generation of precursor metabolites & energy | 0.0000 | 0.0000 |
| M | 3 | BP | GO:0044262 cellular carbohydrate metabolic process | 0.0000 | 0.0001 |
| M | 3 | BP | GO:0006066 alcohol metabolic process | 0.0000 | 0.0004 |
| M | 3 | BP | GO:0006118 electron transport | 0.0002 | 0.0030 |
| M | 3 | BP | GO:0006119 oxidative phosphorylation | 0.0002 | 0.0032 |
| M | 3 | BP | GO:0051186 cofactor metabolic process | 0.0010 | 0.0181 |
| M | 3 | BP | GO:0006006 glucose metabolic process | 0.0011 | 0.0186 |
| M | 3 | BP | GO:0006629 lipid metabolic process | 0.0013 | 0.0236 |
| M | 3 | BP | GO:0006732 coenzyme metabolic process | 0.0019 | 0.0340 |
| M | 3 | BP | GO:0046164 alcohol catabolic process | 0.0026 | 0.0447 |
| M | 3 | BP | GO:0009056 catabolic process | 0.0027 | 0.0466 |
| M | 3 | BP | GO:0007591 molting cycle, chitin-based cuticle | 0.0056 | 0.0940 |
| M | 3 | BP | GO:0019320 hexose catabolic process | 0.0060 | 0.1019 |
| M | 3 | BP | GO:0046365 monosaccharide catabolic process | 0.0060 | 0.1019 |
| M | 3 | BP | GO:0006007 glucose catabolic process | 0.0060 | 0.1019 |
| M | 3 | BP | GO:0005996 monosaccharide metabolic process | 0.0062 | 0.1039 |
| M | 3 | BP | GO:0046034 ATP metabolic process | 0.0062 | 0.1047 |
| M | 3 | BP | GO:0044248 cellular catabolic process | 0.0065 | 0.1095 |
| M | 3 | BP | GO:0019318 hexose metabolic process | 0.0070 | 0.1168 |
| M | 3 | BP | GO:0006508 proteolysis | 0.0078 | 0.1294 |
| M | 3 | BP | GO:0009150 purine ribonucleotide metabolic process | 0.0080 | 0.1334 |
| M | 3 | BP | GO:0019751 polyol metabolic process | 0.0085 | 0.1402 |
| M | 3 | BP | GO:0006071 glycerol metabolic process | 0.0085 | 0.1402 |
| M | 3 | BP | GO:0016052 carbohydrate catabolic process | 0.0088 | 0.1445 |
| M | 3 | BP | GO:0044275 cellular carbohydrate catabolic process | 0.0088 | 0.1445 |
| M | 3 | MF | GO:0016491 oxidoreductase activity | 0.0000 | 0.0001 |
| M | 3 | MF | GO:0048037 cofactor binding | 0.0001 | 0.0010 |
| M | 3 | MF | GO:0005506 iron ion binding | 0.0003 | 0.0053 |
| M | 3 | MF | GO:0003824 catalytic activity | 0.0005 | 0.0079 |
| M | 3 | MF | GO:0050662 coenzyme binding | 0.0009 | 0.0151 |
| M | 3 | MF | GO:0020037 heme binding | 0.0028 | 0.0447 |
| M | 3 | MF | GO:0046906 tetrapyrrole binding | 0.0028 | 0.0447 |
| M | 3 | MF | GO:0015078 hydrogen ion transmembrane transporter activity | 0.0029 | 0.0467 |
| M | 3 | MF | GO:0015077 monovalent inorganic cation transmembrane transporter activity | 0.0029 | 0.0467 |
| M | 3 | MF | GO:0008236 serine-type peptidase activity | 0.0031 | 0.0490 |
| M | 3 | MF | GO:0017171 serine hydrolase activity | 0.0031 | 0.0490 |
| M | 3 | MF | GO:0008233 peptidase activity | 0.0039 | 0.0616 |
| M | 3 | MF | GO:0009055 electron carrier activity | 0.0040 | 0.0632 |
| M | 3 | MF | GO:0004497 monooxygenase activity | 0.0042 | 0.0675 |
| M | 3 | MF | GO:0022890 inorganic cation transmembrane transporter activity | 0.0056 | 0.0877 |
